# Checkpoint inhibitor therapy in preclinical sepsis models: a systematic review and meta-analysis

**DOI:** 10.1186/s40635-019-0290-x

**Published:** 2020-02-04

**Authors:** Lindsay M. Busch, Junfeng Sun, Xizhong Cui, Peter Q. Eichacker, Parizad Torabi-Parizi

**Affiliations:** Critical Care Medicine Department, National Institutes of Health Clinical Center, Bethesda, USA

**Keywords:** Checkpoint molecule, Checkpoint inhibitor, Sepsis, Preclinical model, Bacterial infection, Treatment

## Abstract

**Background:**

Animal studies reporting immune checkpoint inhibitors (CPIs) improved host defense and survival during bacterial sepsis provided one basis for phase I CPI sepsis trials. We performed a systematic review and meta-analysis examining the benefit of CPI therapy in preclinical studies, and whether variables potentially altering this clinical benefit were investigated. Studies were analyzed that compared survival following bacteria or lipopolysaccharide challenge in animals treated with inhibitors to programmed death-1 (PD-1), PD-ligand1 (PD-L1), cytotoxic T lymphocyte-associated protein-4 (CTLA-4), or B- and T-lymphocyte attenuator (BTLA) versus control.

**Results:**

Nineteen experiments from 11 studies (*n* = 709) were included. All experiments were in mice, and 10 of the 19 were published from a single research group. Sample size calculations and randomization were not reported in any studies, and blinding procedures were reported in just 1. Across all 19 experiments, CPIs increased the odds ratio for survival (OR, 95% CI) [3.37(1. 55, 7.31)] but with heterogeneity (*I*^2^ = 59%, *p* < 0.01). After stratification by checkpoint molecule targeted, challenge site or type, or concurrent antibacterial treatment, CPIs had consistent effects over most experiments in the 9 that included antibacterial treatment [OR = 2.82 (1.60, 4.98), *I*^2^ = 6%, *p* = 0.39 with versus 4.01 (0.89, 18.05), *I*^2^ = 74%, *p* < 0.01 without]. All 9 antibiotic experiments employed cecal-ligation and puncture (CLP) bacterial challenge while 6 also included a *Candida albicans* challenge 3–4 days after CLP. In these six experiments (*n* = 322), CPIs were directed at the fungal challenge when CLP lethality had resolved, and were consistently beneficial [2.91 (2.41, 3.50), *I*^2^ = 0%, *p* = 0.99]. In the three experiments (*n* = 66) providing antibiotics without fungal challenge, CPIs were administered within 1 day of CLP and had variable and non-significant effects [0.05 (0.00, 1.03); 7.86 (0.28, 217.11); and 8.50 (0.90, 80.03)]. No experiment examined pneumonia.

**Conclusions:**

Preclinical studies showing that CPIs add benefit to antibiotic therapy for the common bacterial infections causing sepsis clinically are needed to support this therapeutic approach. Studies should be reproducible across multiple laboratories and include procedures to reduce the risk of bias.

## Background

Immune checkpoint molecules regulate T lymphocyte function [1] and when activated, reduce T cell proliferation, inflammatory cytokine production, and longevity [2, 3]. Checkpoint inhibitors (CPIs) can sustain lymphocyte activation and benefit host defense in select clinical contexts. Monoclonal antibodies (mAb) blocking programmed cell death-1 (PD-1), its ligand PD-L1, or cytotoxic T lymphocyte-associated protein-4 (CTLA-4) augment tumor-reactive cytotoxic T cell function and are currently FDA-approved treatments for several cancers [4, 5]. Inhibition of PD-1 and PD-L1 has also been shown to improve pathogen clearance in some viral and protozoal infection models [6].

Checkpoint molecule expression is reportedly increased in septic patients, and there has been interest in using CPIs to augment host defense for sepsis due to acute bacterial infection [7, 8]. This therapeutic approach has risks though, since CPIs could elicit host inflammation and aggravate sepsis-associated inflammatory injury [7]. Despite such risks, CPIs have been reported to improve bacterial clearance and survival in several animal bacterial sepsis models [9–12]. Based in part on these preclinical studies, two phase I clinical trials have been conducted testing CPI therapy in patients presenting with severe sepsis or septic shock [13, 14]. However in one, treatment with an anti-PD-L1 mAb did not have apparent benefit, and a planned phase II trial was not conducted [13]. A second phase I sepsis trial of an anti-PD-1 mAb was completed in January 2018, but further clinical trials have not been announced [14].

While these preclinical and clinical experiences with CPIs appear at odds, the sepsis field has been characterized by immunomodulator agents that were reportedly beneficial in early animal studies but failed in subsequent clinical trials. Several factors have been cited to explain these differing results. While the site and type of infection are uniform for subjects in animal studies, these vary in patients. An immunomodulator beneficial under one set of conditions might be ineffective or even harmful under another [15–17]. Also, while antibiotics are standard clinically, they are frequently not included in animal sepsis models where their absence might favor immunomodulator agents, especially ones augmenting host defense [18]. The cardiopulmonary support patients receive, which could also negate an immunomodulator’s benefit, is also rarely used in animal models [18, 19]. Finally, lack of sample size calculations and randomization and blinding procedures standard in clinical trials and the tendency to publish positive but not negative results may bias preclinical reports [20].

We sought to better understand whether factors like those cited above may have contributed to discrepant results between published animal studies and the present small clinical experience with CPIs in sepsis. We performed a systematic review and meta-analysis of published preclinical sepsis studies comparing survival in bacteria- or lipopolysaccharide (LPS)-challenged groups receiving CPIs versus control. We hypothesized that overall published animal sepsis studies would report benefit with CPI, but would not account for variables potentially influencing these agents’ purported benefits.

## Methods

This systematic review was registered with PROSPERO on October 15, 2018 (CRD42018109798), and prepared using the Preferred Reporting Items for Systematic Reviews and Meta-Analyses (PRISMA) statement for literature review and data extraction (Additional file 1: Appendix 1).

### Literature search and study selection

Using guidelines [21] and search strategies presented in Additional file 1: Appendix 2, two authors (LMB, PTP) identified relevant studies in PubMed, EMBASE, Scopus, and Web of Science from inception through March 13, 2019, without language restrictions. Included studies were searched for additional references. Studies were analyzed if they included experiments comparing the effect of an inhibitor of PD-1, PD-L1, CTLA-4, or BTLA to a control agent on survival following a bacterial or LPS challenge. Studies employing a non-bacterial challenge in addition to bacterial or LPS challenge were included.

### Data extracted and outcomes examined

Study data were extracted by two investigators (LMB, PQE) using a standardized tool including animal number, species, strain, age, and sex; type and site of bacterial or LPS challenge; type and timing of additional challenges if used; type and regimen of CPI investigated; type and regimen of antimicrobial or other supportive treatments; and observation duration. The day of bacterial challenge was designated day 0 (D0), and other interventions were recorded relative to D0.

The primary outcome examined was the effect of CPI treatment on the odds ratio (95% CI) of survival (OR) based on the number of animals reported living at the end of observation periods. In studies including more than one experiment, each experiment was analyzed individually. In studies employing a nonspecific antibody or other protein treatment in one control group and a saline or other diluent in another, the former control group was analyzed. When survival was only provided with Kaplan-Meier plots, study authors were contacted to obtain animal numbers contributing to the plotted outcomes. If the authors did not respond, survival rates were estimated from the Kaplan-Meier plots independently by two authors (LMB, PQE) and agreed to by consensus.

Secondary outcomes assessed included the effect of infectious challenge on the checkpoint molecule targeted and the effect of CPI on bacterial counts, organ injury, cytokine levels, cytokine production by isolated cells tested ex vivo, immune cell numbers in blood or tissues, and apoptosis. Data were extracted from experiments that employed similar challenges and treatment regimens as in survival experiments and only if statistical analysis comparing CPI and control groups was provided.

Risk of bias was assessed in studies based on a modified version of the Systemic Review Centre for Laboratory Animal Experimentation (SYRCLE) grading system and whether studies reported sample size calculations; randomization of challenges and treatments; blinding of challenges, treatments, and survival assessment; confirmation of the baseline similarity of study group animals (e.g., weight, age); removal of animals during study; and randomized housing [22, 23].

### Statistical analysis

The odds ratio of survival with CPI versus the control group was estimated using a random-effects model [24] and the Knapp-Hartung adjustment for small study numbers [25]. The effects of CPI treatment on survival were prospectively planned to be analyzed based on four variables including the checkpoint molecule targeted; site and type of bacterial challenge; and inclusion of anti-bacterial treatment. In one study in which CPI therapy was administered 1 h before or 1 or 3 h after LPS challenge in three different treatment groups, these groups were combined and compared to the single control group [26]. In one study [11], a common control group was used for two experiments. We split the control group data evenly so they are not used twice in our analysis. Heterogeneity among studies was assessed using the *Q*-statistic and *I*^2^ value and was considered moderate or greater for *I*^2^ ≥ 35% [27]. Due to the differing assays and tissues sampled across studies for secondary outcome data, these results were summarized and presented as qualitative differences between CPI versus control groups (i.e., statistically significantly increased or decreased or not significantly different). Publication bias was assessed by funnel plot and Egger’s regression [28]. All analyses were performed using R (version 3.6.0) packages *meta* (version 4.9-5) and *metafor* (version 2.1-0) [29–31]. Two-sided *p* values ≤ 0.05 were considered significant.

## Results

### Summary of studies and experiments analyzed

Of 1565 retrieved reports, 11 studies with 19 experiments met the inclusion criteria (Additional file 1: Figure S1) [11, 12, 26, 32–39]. These experiments were all conducted in mice and were analyzed individually. Tables 1 and 2 summarize for each experiment the type and timing of CPI therapy, the bacterial and non-bacterial challenges administered, whether and how antibacterial or other treatments were employed, and the numbers of total animals and survivors. Overall, the 19 experiments included 338 control and 371 CPI-treated animals. Importantly, of the 19 included experiments, 10 were published from the same laboratory. Additionally, assessment for risk of bias revealed that nearly all of the domains included in the SYRCLE tool were not reported, except for one study which did report blinding to treatment (Table 3).

**Table 1:** Overview of checkpoint molecules (CPM) targeted, mouse strains studied, bacterial and additional challenges employed, and the number of total and surviving animals in control and inhibitor treatment groups in each experiment analyzed from the retrieved studies

Author, year	Exp ID	CPM target	Mouse strain	Bacterial challenge			Additional challenge	Animal numbers			
				Organism	Site	Abx Rx		Control total	Control survivors	Inhibitor total	Inhibitor survivors
Seo, 2008	1	PD-L1	C57BL6	*L. monocytogenes*	IV	No	None	10	10	10	3
Zhang, 2010*	1	PD-L1	C57BL6	CLP—Polymicrobial	IP	No	None	12	2	18	13
	2	PD-L1	C57BL6	CLP—Polymicrobial	IP	No	None	12	2	18	9
Kobayashi, 2013	1	BTLA	C57BL6	LPS	IP	No	None	10	0	30	23
Cheng, 2016	1	BTLA	C57BL6	CLP—Polymicrobial	IP	No	Hemorrhage—pre	15	11	15	5
Deng, 2018^#^	1	PD-L1	C57BL6	CLP—Polymicrobial	IP	No	None	20	11	20	18
	2	PD-L1	Bmal-/-	CLP—Polymicrobial	IP	No	None	20	5	20	14
Patil, 2018	1	PD-L1	BALB/c	*P. aeruginosa*	ID	No	Skin Burn—pre	15	2	15	10
	2	PD-L1	BALB/c	*S. aureus*	IV	No	Skin Burn—pre	15	2	15	8
Inoue, 2011**	1	CTLA-4	CD1	CLP—Polymicrobial	IP	Yes	None	18	1	18	6
	2	CTLA-4	CD1	CLP—Polymicrobial	IP	Yes	None	10	5	10	0
	3	CTLA-4	C57BL6	CLP—Polymicrobial	IP	Yes	None	5	0	5	2
	4	CTLA-4	CD1	CLP—Polymicrobial	IP	Yes	IV Candida—post	7	0	7	2
Chang, 2013	1	PD-1	C57BL6 or CD1	CLP—Polymicrobial	IP	Yes	IV Candida—post	74^@^	25	35	20
	2	PD-L1	C57BL6 or CD1	CLP—Polymicrobial	IP	Yes	IV Candida—post	74^@^	25	39	23
	3	CTLA-4	C57BL6 or CD1	CLP—Polymicrobial	IP	Yes	IV Candida—post	18	6	19	11
Shindo, 2015	1	PD-1	C57BL6	CLP—Polymicrobial	IP	Yes	IV Candida—post	30	25	28	26
Shindo, 2017	1	PD-L1	CD1	CLP—Polymicrobial	IP	Yes	IV Candida—post	33	10	32	19
Brahmamdam, 2010	1	PD-1	CD1	CLP—Polymicrobial	IP	No	None	14	4	17	12

See Table 2 for additional details regarding challenge and treatment regimens

*Exp ID* number assigned the experiment(s) providing survival data in each study, *CPM* checkpoint molecule targeted, *PD-1* programmed cell death 1, *PD-L1* programmed cell death ligand-1, *CTLA-4* cytotoxic T lymphocyte-associated protein-4, *BTLA* B and T lymphocyte attenuator, *Abx Rx* antibiotic treatment, *CLP* cecal ligation and puncture, *IV* intravenous, *IP* intraperitoneal, *ID* intradermal, *post* additional challenge administered after bacterial challenge, *pre* additional challenge administered before bacterial challenge, *LPS* lipopolysaccharide

*Checkpoint inhibitor treatment administered at D−1 in experiment 1 and D0 in experiment 2

**Experiment 1 administered 50 μg and experiment 2 administered 200 μg anti-CTLA-4 in CD-1 mice, experiment 3 administered 50 μg anti-CTLA-4 in C57BL6 mice

^#^Experiment 1 performed in C57BL6J mice and experiment 2 performed in Bmal1^Mye-/-^ mice

^@^A common control group used for these two experiments

**Table 2:** Overview of checkpoint inhibitor regimen, bacterial and non-bacterial challenges, and antibiotic regimen in each experiment analyzed from the retrieved studies

Study (author, year)	Exp ID	Checkpoint inhibitor regimen*				Bacterial challenge**					Additional non-bacterial challenge						
						Challenge regimen			Antibiotic regimen		Challenge regimen				Antimicrobial regimen		
		Target	Time^@^	Dose	Route	Type	Site	Dose	Type	Time^@^	Type	Time^@^	Site	Dose	Type	Time^@^	Route
Seo, 2008	1	PD-L1	D-1	200 μg	IP	*L. mono*	IV	30000 CFU	NR	NR	NA						
Zhang, 2010	1	PD-L1	D-1	50 μg	IP	CLP	IP	NA	NR	NR	NA						
	2	PD-L1	D0^&^	50 μg	IP	CLP	IP	NA	NR	NR	NA						
Kobayashi, 2013	1	BTLA^#^	D0^^	400 μg	IP	LPS	IP	750 μg	NA	NA	NA						
Cheng, 2016	1	BTLA^#^	D-1	25 μg/g	IV	CLP	IP	NA	NR	NR	Hem	D-1	NA				
			D0	25 μg/g	IP												
Deng, 2018	1	PD-L1	D0,+1,+2,+3^@@^	20 mg/kg	NR	CLP	IP	NA	NR	NR	NA						
	2	PD-L1	D0,+1,+2,+3^@@^	20 mg/kg	NR	CLP	IP	NA	NR	NR	NA						
Patil, 2018	1	PD-L1	D-1	50 μg	IP	*P. aer*	ID	1x10^6^ CFU	NR	NR	Burn	D-4	Skin	NA			
	2	PD-L1	D-1	200 μg	IP	*S. aur*	IV	1x10^8^ CFU	NR	NR	Burn	D-4	Skin	NA			
Brahmamdam, 2010	1	PD-1	D+1, +2	200 μg	IV	CLP	IP	NA	NR	NR	NA						
Inoue, 2011	1	CTLA-4	D0,+1^@@, &&^	50 μg	IP	CLP	IP	NA	Imi	D0	NA						
	2	CTLA-4	D0, +1	200 μg	IP	CLP	IP	NA	Imi	D0	NA						
	3	CTLA-4	D0,+1, +2^@@^	50 μg	IP	CLP	IP	NA	Imi	D0	NA						
	4	CTLA-4	D+6,+9,+11	33 μg	IP	CLP	IP	NA	Imi	D0	*C. alb*	D+4	IV	UC	NR	NR	NR
Chang, 2013	1	PD-1	D+5,+8,+11	200 μg	IP	CLP	IP	NA	Imi	D+1	*C. alb*	D+3	IV	UC	Fluc	D+9-12	IP
	2	PD-L1	D+5,+8,+11	200 μg	IP	CLP	IP	NA	Imi	D+1	*C. alb*	D+3	IV	UC	Fluc	D+9-12	IP
	3	CTLA-4	D+4	100 μg	IP	CLP	IP	NA	Imi	D+1	*C. alb*	D+3	IV	UC	Fluc	D+9-12	IP
Shindo, 2015	1	PD-1	D+4,+8	200 μg	IP	CLP	IP	NA	Imi	D0	C. alb	D+3	IV	UC	Fluc	D+5,+6	IP
Shindo, 2017	1	PD-L1^	D+5 to D+13 (TID)	3 mg/kg	SC	CLP	IP	NA	Imi	D0	*C. alb*	D+3	IV	UC	NR	NR	NR

*Exp ID* experiment identification number within a study, *PD-1* programmed cell death 1, *PD-L1* programmed cell death ligand-1, *CTLA-4* cytotoxic T lymphocyte-associated protein-4, *BTLA* B and T lymphocyte attenuator, *ID* intradermal, *IP* intraperitoneal, *D* day, *L. mono L. monocytogenes*, *P. aer Pseudomonas aeruginosa*, *S. aur Staphylococcus aureus*, *IV* intravenous, *SC* subcutaneous, *CFU* colony-forming unit, *NR* not reported, *NA* not applicable, *CLP* cecal ligation and puncture, *C. alb Candida albicans*, *Imi* imipenem 1 mg total or 2.5 mg/kg administered subcutaneously, *UC* unclear, *Fluc* fluconazole 200 μg, *TID* dose administered 3 times daily, *Hem* hemorrhage

*All CPIs were monoclonal antibodies except Shindo 2017 (^), which employed a peptide inhibitor

^#^The antibody targeting BTLA has been suggested to have both agonistic and antagonistic properties

**Bacterial challenge was designated time 0 (D0) in all experiments

^@^Time for all treatments and additional challenges in reference to the bacterial challenge at D0

^@@^Experiments 1 and 3 in Inoue 2011 performed in CD1 and C57BL6 mouse strains respectively and experiments 1 and 2 in Deng 2018 performed in C57BL6 and Bmal-/- mice respectively

^^Animals treated 30 min before, at the time of, or 30 min after LPS challenge were combined for analysis, see the “Methods” section

^&^CPI treatment was 3 h after CLP

^&&^CPI treatment was 6 h after CLP

**Table 3:** Risk of bias assessment, adapted from SYRCLE

Author, year	Sample size calculation	Randomization procedure	Groups similar at baseline (weight, age)	Blinding to challenge	Blinding to treatment	Blinding to survival assessment	Animals removed from study	Random animal housing
Seo, 2008	NR	NR	NR	NR	NR	NR	NR	NR
Brahmamdam, 2010	NR	NR	NR	NR	NR	NR	NR	NR
Zhang, 2010	NR	NR	NR	NR	NR	NR	NR	NR
Inoue, 2011	NR	NR	NR	NR	NR	NR	NR	NR
Chang, 2013	NR	NR	NR	NR	NR	NR	NR	NR
Kobayashi, 2013	NR	NR	NR	NR	NR	NR	NR	NR
Shindo, 2015	NR	NR	NR	NR	NR	NR	NR	NR
Cheng, 2016	NR	NR	NR	NR	NR	NR	NR	NR
Shindo, 2017	NR	NR	NR	NR	Yes	NR	NR	NR
Deng, 2018	NR	NR	NR	NR	NR	NR	NR	NR
Patil, 2018	NR	NR	NR	NR	NR	NR	NR	NR

*NR* not reported, *SYRCLE* Systematic Review Center for Laboratory Experimentation

Checkpoint inhibitors were directed against PD-L1 in nine experiments, PD-1 in three, CTLA-4 in five, and BTLA in two (referred to subsequently as anti-PD-1, anti-PD-L1, anti-CTLA-4, and anti-BTLA when required). All studies employed mAbs, except for one study testing a peptide inhibitor of PD-L1 [37]. Several studies have suggested that the mAb clone used to target BTLA in the two experiments analyzed, while antagonistic under some conditions, can be agonistic under others [40–43]. All treatments will be referred to as CPIs in the text and figures.

Fifteen experiments (79%) included cecal-ligation and puncture (CLP) bacterial challenge (i.e., polymicrobial), either alone (*n* = 9) or followed 3 to 4 days later by intravenous *Candida albicans* challenge (*n* = 6). One CLP model included hemorrhage challenge 1 day before CLP. Of the four experiments not employing CLP, one administered *Listeria monocytogenes* intravenously (IV), one LPS IP, and two either *Pseudomonas aeruginosa* intradermally (ID) or *Staphylococcus aureus* IV 4 days after skin burn. No experiment examined bacterial pneumonia.

In experiments with bacterial (including CLP) or LPS challenge alone, CPI treatment was administered starting 1 day before, on the day of, or 1 day after challenge (D−1, D0, or D+1 in Table 2). In all six experiments with CLP followed 3 to 4 days later by *C. albicans*, CPI treatment was started 1 to 2 days following fungal challenge (i.e., 4 to 6 days after CLP) and targeted the fungal infection since CLP mortality is largely resolved in 3 to 4 days.

Nine experiments investigated CPI with antibacterial therapy, and these all included CLP challenge with imipenem treatment. Six of the CLP experiments also included subsequent *C. albicans* challenge, and four of these administered fluconazole with fungal challenge. While all CLP experiments included a 1- to 2-ml subcutaneous normal or phosphate-buffered saline injection following CLP, none included later hemodynamic support. The four non-CLP experiments did not include supportive measures.

### Effect of infectious challenge on the checkpoint molecule targeted in experiments

Twelve experiments from eight studies (Additional file 1: Table S1) examined the effect of bacterial challenge on expression of the checkpoint molecule targeted with CPI therapy in infected versus noninfected untreated animals. In at least 11 experiments, the bacterial challenge increased the expression of the checkpoint molecule targeted (*p* ≤ 0.05). One study reported increased expression, but a *p* value was not provided.

### Effect of checkpoint inhibitor therapy on survival

**Fig. 1:**
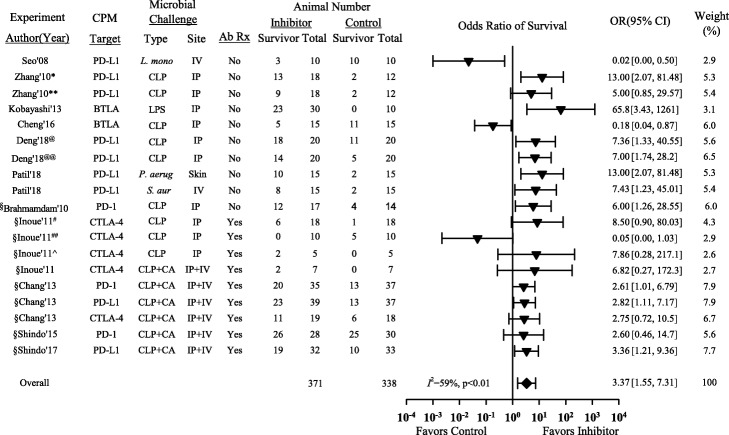
Effects of checkpoint inhibitor (CPI) therapy on the odds ratios of survival (95% CI) in each of the 19 analyzed experiments and the overall OR (95% CI) and its *I*^2^ with level of significance. Shown for each experiment is the checkpoint molecule (CPM) targeted with CPI, the type and site of the bacterial challenge employed, whether a secondary intravenous (IV) *C. albicans* challenge was included, whether antibiotic treatment for the bacterial challenge was administered, and the numbers of total and surviving animals in the control and CPI groups. Checkpoint molecule inhibitors increased the odds ratio of survival OR (95% CI) in 16 experiments (10 significantly) and decreased it in 3 (2 significantly). The overall OR was increased with CPI therapy but with heterogeneity. ^§^Experiments from studies published by the same research group; *CPI administered at D−1; **CPI administered at D0; ^#^anti-CTLA-4 50 μg in CD-1 mice; ^##^anti-CTLA-4 200 μg CD-1 mice; ^C57BL6 mice; ^@^C57BL6 mice; ^@@^Bmal-/- mice; Ab Rx—antibiotic treatment for the primary bacterial challenge; CA—*Candida albicans*; PD-1—programmed cell death 1; PD-L1—programmed cell death ligand-1; CTLA-4—cytotoxic T lymphocyte-associated protein-4; BTLA—B and T lymphocyte attenuator; CPM—checkpoint molecule targeted; CLP—cecal ligation and puncture which represented polymicrobial organisms; IV—intravenous; IP—intraperitoneal; skin—intradermal; LPS—lipopolysaccharide; *L. mono*—*Listeria monocytogenes*; *P. aerug—Pseudomonas aeruginosa*; *S. aur—Staphylococcus aureus*

CPIs increased the odds ratio of survival [OR (95% CI)] in 16 experiments (10 significantly) and decreased it in 3 (2 significantly) (Fig. 1). The overall OR was increased with CPI therapy [3.37 (1.55, 7.31)] but with heterogeneity (*I*^2^ = 59%, *p* < 0.01). The three experiments in which CPI treatment had an effect on the side of harm included treatment with a PD-L1 mAb with IV *L. monocytogenes*, a high dose of CTLA-4 mAb with CLP alone, and a BTLA mAb with CLP following hemorrhage challenge.

**Fig. 2:**
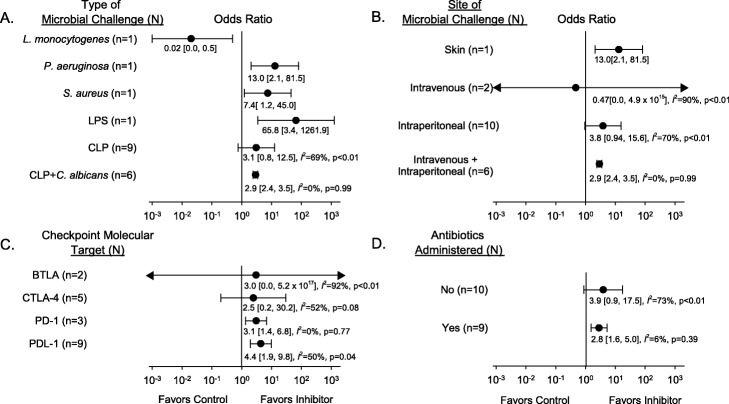
Effects of checkpoint molecule inhibitor (CPI) therapy on the overall odds ratios of survival (95% CI) and *I*^2^s with levels of significance for each of the subgroups of experiments analyzed. Experiments were analyzed based on the following parameters: the type of microbial challenge (**a**), the site of microbial challenge (**b**), check point molecule targeted (**c**), and whether antibiotic treatment for the bacterial challenge was or was not administered (**d**). Also shown are the number of experiments (*n*) comprising each subgroup. Checkpoint inhibitor therapy had consistent effects over the greatest number of studies in the nine that included antibiotic treatment (*I*^2^ = 6%, *p* = 0.39). CLP—cecal ligation and puncture which represented polymicrobial organisms; LPS—lipopolysaccharide; PD-1—programmed cell death 1; PD-L1—programmed cell death ligand-1; CTLA-4—cytotoxic T lymphocyte associated protein-4; BTLA—B and T lymphocyte attenuator

**Fig. 3:**
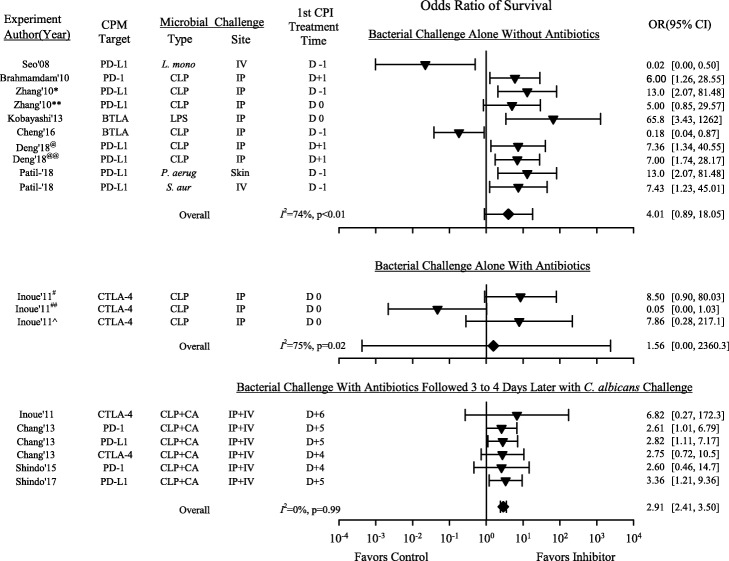
Effects of checkpoint inhibitor (CPI) therapy on the odds ratios of survival (95% CI) in each of the 19 analyzed experiments in three subgroups with its *I*^2^ and level of significance. The subgroups are based on whether experiments included a bacterial challenge alone without antibiotic therapy, a bacterial challenge alone with antibiotic therapy directed at the bacterial challenge or a bacterial challenge with antibiotic therapy directed at the bacterial challenge followed by a *C. albicans* challenge 3 to 4 days later, as well as the overall OR and its *I*^2^ with level of significance for each subgroup. Also shown are the times the CPI therapy was administered relative to the bacterial challenge. *CPI administered at D−1; **CPI administered at D0; ^#^anti-CTLA-4 50 μg in CD-1 mice; ^##^anti-CTLA-4 200 μg CD-1 mice; ^C57BL6 mice; ^@^C57BL6 mice; ^@@^Bmal-/- mice. CA—*Candida albicans*; PD-1—programmed cell death 1; PD-L1—programmed cell death ligand-1; CTLA-4—cytotoxic T lymphocyte-associated protein-4; BTLA—B and T lymphocyte attenuator; CPM—checkpoint molecule targeted; CLP—cecal ligation and puncture which represented polymicrobial organisms; IV—intravenous; IP—intraperitoneal; LPS—lipopolysaccharide; *L. mono*—*Listeria monocytogenes*; *P. aerug—Pseudomonas aeruginosa*; *S. aur—Staphylococcus aureus*

When experiments were stratified by the type of checkpoint molecule targeted, site or type of infectious challenge, or presence of antibacterial treatment, CPI treatment had consistent effects over the greatest number of studies in the nine that included antibacterial treatment (Fig. 2). In the 10 experiments (*n* = 321 total animals) without antibacterial therapy, CPIs increased the OR in eight (seven significantly) in a pattern approaching significance but with heterogeneity [4.01 (0.89, 18.05); *I*^2^ = 74%, *p* < 0.01] (Fig. 3). In the nine experiments with antibacterial therapy (*n* = 388 total animals), all with CLP challenge, CPI increased overall survival significantly and consistently [2.82 (1.60, 4.98); *I*^2^ = 6%, *p* = 0.39] (Fig. 2). However, these nine experiments only included three with CLP alone (*n* = 66 total animals) but six with CLP followed 3 to 4 days later by *C. albicans* challenge (*n* = 322 total animals). While CPI therapy was administered within 1 day of CLP in the former three experiments, it was administered following the fungal challenge in the six other experiments when lethality related to CLP would have largely resolved (i.e., 3 to 4 days after CLP); therefore, these two groups of experiments were also analyzed separately (Fig. 3). CPI administration after the fungal challenge had highly consistent beneficial effects [2.91 (2.41, 3.50); *I*^2^ = 0%, *p* = 0.99]. However, in the only three experiments in this analysis which tested CPI and antibacterial therapy together with bacterial challenge alone, CPIs had highly variable effects on the ORs [0.05 (0.00, 1.03); 7.86 (0.28, 217.11); and 8.5 (0.90, 80.03)], and the overall survival effect was far from significant [1.56 (0.00, 2360.31); *I*^2^ = 75%, *p* = 0.02].

Funnel plot and Egger’s statistic (*p* = 0.96) suggested that the overall survival results were not subject to publication bias (Additional file 1: Figure S2). However, none of the 11 studies reported sample size calculations, random allocation of animals to challenges, treatments, or housing or blinding of survival assessment. Only 1 study reported blinding of treatment. Overall risk of bias was unclear in all studies (Table 3).

### Effect of checkpoint inhibitor treatment on microbial clearance

As summarized in Table 4, seven experiments reported the effects of CPI treatment on quantitative bacterial cultures. None of the seven included antibiotic therapy. In three CLP experiments and one with ID *P. aeruginosa* challenge, anti-PD-L1 decreased bacterial counts in blood, peritoneal fluid, or lung tissue on D+1, D+2, or D+3 (*p* ≤ 0.05). Anti-BTLA had no significant effect (*p* = ns) on blood and peritoneal bacterial counts in one CLP experiment, and anti-PD-L1 had no significant effect on lung or spleen bacterial counts with IV *S. aureus* in another*.* Finally, anti-PD-L1 increased liver and spleen bacterial counts on D+3 following IV *L. monocytogenes* (*p* ≤ 0.05).

**Table 4:** Effect of checkpoint inhibitor therapy on blood and tissue bacterial counts and organ injury measures

Author, year	Exp ID	Checkpoint molecule target	Challenge	Bacterial counts			Organ injury		
				Decrease	Increase	No difference	Decrease	Increase	No difference
Models not including antibacterial agents									
Seo, 2008	1	PD-L1	IV *L. mono*	–	Spleen, liver D+3	–	NR	NR	NR
Brahmamdam, 2010	1	PD-1	CLP	NR	NR	NR	NR	NR	NR
Zhang, 2010*	1	PD-L1 (D−1)	CLP	NR	NR	NR	NR	NR	NR
	2	PD-L1 (D0)	CLP	Peritoneal, blood D+1	–	–	NR	NR	NR
Kobayashi, 2013	1	BTLA	LPS	NA	NA	NA	NR	NR	NR
Cheng, 2016	1	BTLA	CLP	–	–	Peritoneal, blood D+1	–	Lung, kidney D+1	Liver D+1
Deng, 2018^#^	1	PD-L1	CLP	Peritoneal, blood D+1, D+2, D+3	–	–	Lung, kidney, liver, muscle, intestine D+1, D+2, D+3	–	–
	2	PD-L1	CLP	Peritoneal, blood D+1, D+2, D+3	–	–	Lung, kidney, liver, muscle, intestine D+1, D+2, D+3	–	–
Patil, 2018	1	PD-L1	ID *P. aer*	Lung, blood D+2	–	–	Kidney, liver D+2	–	–
	2	PD-L1	IV *S. aur*	–	–	Lung, spleen D+3	Kidney, liver D+3	–	–
Models including antibacterial agents									
Inoue, 2011 **	1	CTLA-4	CLP	NR	NR	NR	NR	NR	NR
	2	CTLA-4	CLP	NR	NR	NR	NR	NR	NR
	3	CTLA-4	CLP	NR	NR	NR	NR	NR	NR
	4	CTLA-4	CLP + IV *C.a.*	NR	NR	NR	NR	NR	NR
Chang, 2013	1	PD-1	CLP + IV *C.a.*	NR	NR	NR	NR	NR	NR
	2	PD-L1	CLP + IV *C.a.*	NR	NR	NR	NR	NR	NR
	3	CTLA-4	CLP + IV *C.a.*	NR	NR	NR	NR	NR	NR
Shindo, 2015	1	PD-1	CLP + IV *C.a.*	NR	NR	NR	NR	NR	NR
Shindo, 2017	1	PD-L1	CLP + IV *C.a.*	NR	NR	NR	NR	NR	NR

*Exp ID* number assigned the experiment(s) providing survival data in each study, *C.a. Candida albicans*, *L. mono Listeria monocytogenes*, *S. aur Staphylococcus aureus*, *P. aer Pseudomonas aeruginosa*, *PD-L1* programmed death ligand-1, *PD-1* programmed cell death-1, *CTLA-4* cytotoxic T lymphocyte-associated protein-4, *BTLA* B and T lymphocyte attenuator, *CLP* cecal ligation and puncture, *D* day, *ID* intradermal, *IV* intravenous, *IP* intraperitoneal, *NR* not reported, *NA* not applicable

*Checkpoint inhibitor administered at D−1 in exp 1 and D0 in exp 2

**Exp 1 administered 50 μg and exp 2 administered 200 μg anti-CTLA-4 in CD-1 mice, exp 3 administered 50 μg anti-CTLA-4 in C57BL6 mice

^#^Exp 1 performed in C57BL6J mice and exp 2 in Bmal1^Mye-/-^ mice

^##^IV *Candida* challenge 4 days following CLP

### Effect of checkpoint inhibitor treatment on organ injury

Five experiments, none including antibiotics, provided data regarding the effects of CPI on organ injury (Table 4). Lung, liver, renal, intestinal, and/or muscle injury as reflected by changes in lung lavage protein, serum creatinine, blood urea nitrogen levels, serum alanine or aspartate aminotransferase levels, intestinal histology, or creatine phosphokinase levels were decreased with anti-PD-L1 treatment in four experiments on D+1 to D+3 after either CLP, ID *P. aeruginosa*, or IV *S. aureus* (all *p* ≤ 0.05). However, lung lavage protein and serum creatinine levels were increased with anti-BTLA treatment on D+1 after CLP (*p* = 0.05).

### Effect of checkpoint inhibitor treatment on serum and tissue cytokines, immune cell populations, and apoptosis

The effects of CPI treatment on serum or tissue cytokines, immune cell populations, and apoptosis were reported in five, eight, nine, and seven experiments, respectively. These results are summarized in Table 5 and described in detail in a supplemental section (Additional file 1: Appendix 3). The effects of CPI treatment on serum and tissue cytokine levels were variable, and no clear pattern was evident. However, possibly consistent with CPI treatment’s proposed pro-inflammatory effects, in one study, anti-PD-L1 treatment was associated with increases in TNFα and IL-6 levels, and a concomitant decrease in IL-10 [12]. Checkpoint inhibitor treatment increased the numbers or activation state of at least one immune cell population in seven experiments but decreased the population examined in one and had no effect in another. Finally, CPI treatment decreased immune cell apoptosis in five studies but had no effect in two.

**Table 5:** Effect of checkpoint inhibitor treatment on serum and tissue cytokines, immune cell populations, and apoptosis

Author, year	Exp ID	Checkpoint molecule target	Infection organism	Infection site	Summary of the effect of checkpoint inhibitor treatment compared to control treatment			
					Serum cytokines	Other cytokines	Cell populations	Apoptosis
Seo, 2008	1	PD-L1	*L. mono*	IV	NR	Heat-killed LM-stim splenocyte TNFα, IL-12p40 and NO production and NK cell IFNγ production decreased on D+3^	Spleen LM-specific CD8s and IFNγ+ CD8s decreased on D+7 and +25^	Frequency of annexin V+ CD8s unchanged at 6 or 24 h
Brahmamdam, 2010	1	PD-1	CLP	IP	IL-6, IL-10, TNFα, IFNγ not different on D+1^ns^	CD3/CD28 stim splenocyte IL-6 production increased on D+2^ but IL-10, TNFα, and IFNγ not different^ns^	Total splenocytes, CD4, CD8, B cells, NK cells, and DCs increased on D+2^^	Splenic CD3 apoptosis decreased on D+2^^
Zhang, 2010*	1	PD-L1	CLP	IP	NR	NR	NR	NR
	2	PD-L1	CLP	IP	TNFα and IL-6 increased, IL-10 decreased on D+1^	NR	Total cell numbers, CD3, and CD19 cell numbers increased on D+1 in blood, spleen, and thymus^	Splenic and thymus lymphocyte apoptosis decreased on D+1^
Inoue, 2011**	1	CTLA-4	CLP	IP	TNFα, IL-6, IL-10, IFNγ not different on D+2^ns^	CD3/CD28 stim splenocyte IL-6, IL-10, TNFα, IFNγ production not different on D+2^ns^	Total splenocyte, CD4, and CD8 numbers unchanged at D+7^ns^. Naïve, effector memory and central memory CD4 and CD8 unchanged at D+7^ns^	Splenic CD4 and CD8 apoptosis decreased on D+2^
	2	CTLA-4	CLP	IP	NR	NR	NR	NR
	3	CTLA-4	CLP	IP	NR	NR	NR	NR
	4	CTLA-4	CLP (*C.a.* D+4)^##^	IP and IV	NR	NR	NR	NR
Kobayashi, 2013	1	BTLA	LPS	IV	NR	NR	NR	NR
Chang, 2013	1	PD-1	CLP (*C.a.* D+3)^##^	IP and IV	NR	CD3/CD28 stim splenocyte IFNγ production increased on D+9^	Macrophage and DC MHCII expression increased at D+9^	NR
	2	PD-L1	CLP (*C.a.* D+3)^##^	IP and IV	NR	CD3/CD28 stim splenocyte IFNγ production not different on D+9^ns^ IFNγ producing CD4 and CD8 increased at D+9^	Macrophage and DC MHCII expression increased at D+9^	NR
	3	CTLA-4	CLP (*C.a.* D+3)^##^	IP and IV	NR	NR	NR	NR
Shindo, 2015	1	PD-1	CLP (*C.a.* D+3) ^##^	IP and IV	NR	Splenic NK, CD4, and CD8 intracellular IFNγ increased, cultured splenocyte IFNγ supernatant not different on D+9	CD28 expression on LN CD4 increased on D+9^; CD28 on splenic CD4 and CD8 not different^ns^; splenic and LN macrophage and DC MHCII not different on D+9	NR
Cheng, 2016	1	BTLA	CLP	IP	MIP-2 increased on D+1^ but TNFα, IL-1β, IL-6, IL-10, IL-12, KC, MCP-1 not different^ns^	Peritoneal lavage TNFα, IL-10, IL-12, KC, MIP-2, MCP-1 and peritoneal macrophage LPS-stim TNFα and MIP-2 increased on D+1^; IL-1β, IL-6 not different in peritoneal lavage on D+1	Total peritoneal leukocyte and F4/80, CD11c+ and Gr1+ cells increased on D+1^	Peritoneal total cell and macrophage apoptosis not different on D+1^ns^
Shindo, 2017	1	PD-1, PD-L1	CLP (*C.a.* D+3)^##^	IP and IV	NR	NR	NR	NR
Deng, 2018^#^	1	PD-L1	CLP	IP	NR	NR	NR	Splenic CD4 and CD8 apoptosis decreased on D+2 and D+3^
	2	PD-L1	CLP	IP	NR	NR	NR	Splenic CD4 and CD8 apoptosis decreased on D+2 and D+3^
Patil, 2018	1	PD-L1	*P. aer*	ID	IL-6, IL-10, MIP-2, KC, IL-17 decreased on D+2^	Spleen and LN CD8 IFNγ production increased, LN CD4 IFNγ production decreased on D+2^ but spleen CD4 IFNγ production not different^ns^	Spleen and LN CD4 and CD8 cell counts increased on D+2^. LN B cell numbers increased at D+2^, not different in spleen. CD28 expression on LN CD4+ and CD8+ increased at D+2^	NR
	2	PD-L1	*S. aur*	IV	NR	NR	NR	NR

*Exp ID* number assigned the experiment(s) providing survival data in each study, *C.a. Candida albicans*, *L. mono Listeria monocytogenes*, *S. aur Staphylococcus aureus*, *P. aer Pseudomonas aeruginosa*, *DC* dendritic cell, *LN* lymph node, *PD-1* programmed cell death 1, *PD-L1* programmed death ligand-1, *CTLA-4* cytotoxic T lymphocyte associated protein-4, *BTLA* B and T lymphocyte attenuator, *CLP* cecal ligation and puncture, *ID* intradermal, *IV* intravenous, *IP* intraperitoneal, *D* day, *NR* not reported, *stim* stimulated

^*p* < 0.05; ^^*p* ≤ 0.01; ^ns^*p* = ns

*Exp 1 treatment administered at D−1 and exp 2 treatment administered at D0

**Exp 1 administered 50 μg and exp 2 administered 200 μg anti-CTLA-4 in CD-1 mice, exp 3 administered 50 μg anti-CTLA-4 in C57BL6 mice

^#^Exp 1 performed in C57BL6J mice and exp 2 performed in Bmal1^Mye-/-^ mice

^##^Intravenous *Candida* challenge 4 days following CLP

## Discussion

This systematic review suggests several reasons why CPI treatment appeared beneficial in published preclinical sepsis models but not in the present small clinical experience. It also highlights important methodological issues which must be considered when interpreting the results of these preclinical studies. The finding that over half of the experiments were published from a single laboratory raises an important question about generalizability of these results. Furthermore, all studies lacked sufficient reporting of methods designed to reduce bias, including provision of sample size calculations, randomization, and blinding procedures. The importance of these procedures is well-supported by groups such as SYRCLE and ARRIVE. Just as in clinical trials, the extent to which reliable conclusions can be drawn from preclinical studies that are systematically reviewed and used as support for human trials is dependent upon the rigor of these studies’ investigative methods [22, 44].

While the primary rationale for CPI treatment in sepsis is the augmentation of host defense and microbial clearance, there are actually little published animal data demonstrating that early CPI therapy combined with antibacterial treatment improves outcome following bacterial infection. In the ten experiments without antimicrobial agents, CPI treatment increased survival in a trend approaching significance. Also, in the six experiments with CLP and antibacterial therapy followed 3 to 4 days later by *C. albicans* challenge, CPI therapy targeting the fungal challenge had highly consistent beneficial effects. But in the only three experiments that investigated a bacterial challenge alone with antibacterial therapy (i.e., CLP with imipenem), CPI treatment had highly variable effects and was not significantly beneficial. Notably, these three experiments only included 66 animals and were all conducted in a single study. Additional investigation might have shown a more consistent effect.

Other factors may also explain the differing treatment effects of CPIs when comparing the overall preclinical and limited clinical experience to date. First, no published preclinical experiment investigated pneumonia, where CPI’s risk of augmented inflammatory lung injury might interfere with its potential host defense benefit. Yet pulmonary infection is the most frequent cause of sepsis in medical intensive care units, as it was in the anti-PD-L1 phase I trial [13]. Second, these mouse studies did not include cardiopulmonary support comparable to what patients receive, which could negate the benefit observed with CPI treatment. Third, preclinical models do not typically reflect the variety of comorbidities prevalent in critically-ill populations. Finally, the absence of sample size calculations and randomization and blinding procedures in preclinical studies may have confounded the survival findings with CPI treatment.

When examined in the 19 experiments overall, a basis for CPIs’ purported beneficial survival effect is not clear. Only seven experiments reported whether CPIs affected microbial clearance and none of these included antibacterial treatment which could negate CPIs’ effects. While anti-PD-L1 did improve survival and reduce blood, peritoneal, or lung bacterial counts in four experiments, it had no effect on bacterial counts in two, and actually increased spleen and liver bacterial counts in one experiment with IV *L. monocytogenes* challenge. Several experiments did suggest that CPI enhanced some host defense effects including increased splenic lymphocyte numbers, IFNγ-producing CD4 cells or activated lymphocytes in five experiments, and decreased apoptosis in five.

It is also unclear whether increased survival with CPIs was related to the reduced organ injury since only five experiments provided these data. In four experiments, anti-PD-L1 increased survival and decreased evidence of lung, liver, kidney, and/or intestinal injury with either CLP alone or skin *P. aeruginosa* or intravenous *S. aureus* infection after burn injury. None of these experiments included antibacterial treatment, and in three, as noted above, CPIs were associated with reduced bacterial counts. Thus, while improved host defense with CPIs may have reduced tissue injury, this effect could be negated by antibacterial agents.

Consistent improvements in survival in the six experiments with CLP in which CPIs were administered following a subsequent *C. albicans* challenge (four of which also included antifungal therapy) suggest CPIs might be beneficial with fungal superinfection. However, fungal superinfection in patients presenting with bacterial sepsis and not already severely immunosuppressed is actually uncommon. In 1719 patients presenting with bacterial sepsis, only 32 (1.9%) developed a secondary fungal infection [45].

In three experiments, CPI treatment was associated with worsened survival that was or approached significance (Fig. 1). In one experiment with CLP following hemorrhage, anti-BTLA treatment did not alter bacterial counts but was associated with increased lung and kidney injury. As noted in the results, this study employed an anti-BTLA antibody with possible agonistic rather than antagonistic activity [41, 42]. In the study with IV *L. monocytogenes* challenge, an anti-PD-L1 mAb decreased *L. monocytogenes*-specific CD8 cells and IFNγ+CD8 cells and increased spleen and liver bacterial counts. In this case, it is also possible this CPI actually suppressed host defense [32]. In the last of these three experiments, animals challenged with CLP and treated with an antibacterial agent received a dose of anti-CTLA-4 dose four times greater than the one that was protective in another experiment from the same study. In this study, the larger dose of anti-CTLA-4 may have produced inflammatory injury not present with the lower dose, but this study did not provide data on organ injury or microbial clearance to make this assessment.

The present study has limitations. As noted, the absence of microbiologic and organ injury data in most experiments prevented understanding how CPI treatment improved survival. Also, the lack of data regarding sample size calculations, randomization, and blinding procedures prevented an accurate assessment of risk of bias in all studies. In fact, only one study reported a single blinding procedure, and no study provided methods for randomization or sample size calculations. Additionally, five of the 11 studies and all nine experiments with CLP and antibiotic treatment were conducted by a single research group. Confirmation of the findings from these experiments by other groups would be important.

## Conclusions

In conclusion, the present findings suggest that if CPI therapy continues to be a consideration for early sepsis, there should be additional preclinical investigation showing that it will add benefit and not harm when used in combination with standard antibiotic and other supportive measures. These studies should include the range of bacterial infections that septic patients present with and should be conducted with procedures that limit potential risk of bias.

## Supplementary information

**Additional file 1.** **Appendix 1.** Prisma checklist. **Appendix 2.** Literature search strategy. **Appendix 3.** Effect of checkpoint inhibitor treatment on serum and tissue cytokines, immune cell populations, and apoptosis. **Table 1.** Effect of bacterial challenge on the checkpoint molecules targeted in analyzed experiments. **Figure 1.** Flow chart of literature search and study selection. **Figure 2.** Funnel plot of odds ratio (OR) of survival.

## Data Availability

The datasets used and/or analyzed during the current study are available from the corresponding author on reasonable request.

## Abbreviations

ARRIVE: Animal Research: Reporting of *In Vivo* Experiments
BTLA: B- and T-lymphocyte attenuator
CI: Confidence interval
CLP: Cecal-ligation and puncture
CPIs: Checkpoint inhibitors
CTLA-4: Cytotoxic T-lymphocyte associated protein-4
ID: Intradermally
LPS: Lipopolysaccharide
OR: Odds ratio for survival
PD-1: Programmed death-1
PD-L1: PD-ligand1
PRISMA: Preferred Reporting Items for Systematic Reviews and Meta-Analyses
SYRCLE: Systemic Review Centre for Laboratory Animal Experimentation
