# Supplementary material for: Checkpoint inhibitor therapy in preclinical sepsis models: a systematic review and meta-analysis
Source: Intensive Care Med Exp. 2020 Feb 4;8:7. doi: 10.1186/s40635-019-0290-x (PMC7000606; doi:10.1186/s40635-019-0290-x)

Appendix 1

PRISMA 2009 Checklist

Section/topic	#	Checklist item	Reported on page #
**TITLE**			
Title	1	Identify the report as a systematic review, meta-analysis, or both.	1
**ABSTRACT**			
Structured summary	2	Provide a structured summary including, as applicable: background; objectives; data sources; study eligibility criteria, participants, and interventions; study appraisal and synthesis methods; results; limitations; conclusions and implications of key findings; systematic review registration number.	2-3
**INTRODUCTION**			
Rationale	3	Describe the rationale for the review in the context of what is already known.	4
Objectives	4	Provide an explicit statement of questions being addressed with reference to participants, interventions, comparisons, outcomes, and study design (PICOS).	5
**METHODS**			
Protocol and registration	5	Indicate if a review protocol exists, if and where it can be accessed (e.g., Web address), and, if available, provide registration information including registration number.	6
Eligibility criteria	6	Specify study characteristics (e.g., PICOS, length of follow-up) and report characteristics (e.g., years considered, language, publication status) used as criteria for eligibility, giving rationale.	6
Information sources	7	Describe all information sources (e.g., databases with dates of coverage, contact with study authors to identify additional studies) in the search and date last searched.	6
Search	8	Present full electronic search strategy for at least one database, including any limits used, such that it could be repeated.	Appendix 2
Study selection	9	State the process for selecting studies (i.e., screening, eligibility, included in systematic review, and, if applicable, included in the meta-analysis).	Supplementary figure 1
Data collection process	10	Describe method of data extraction from reports (e.g., piloted forms, independently, in duplicate) and any processes for obtaining and confirming data from investigators.	6
Data items	11	List and define all variables for which data were sought (e.g., PICOS, funding sources) and any assumptions and simplifications made.	6-7
Risk of bias in individual studies	12	Describe methods used for assessing risk of bias of individual studies (including specification of whether this was done at the study or outcome level), and how this information is to be used in any data synthesis.	7
Summary measures	13	State the principal summary measures (e.g., risk ratio, difference in means).	7-8
Synthesis of results	14	Describe the methods of handling data and combining results of studies, if done, including measures of consistency (e.g., I^2) for each meta-analysis.	8

PRISMA 2009 Checklist

Section/topic	#	Checklist item	Reported on page #
Risk of bias across studies	15	Specify any assessment of risk of bias that may affect the cumulative evidence (e.g., publication bias, selective reporting within studies).	7
Additional analyses	16	Describe methods of additional analyses (e.g., sensitivity or subgroup analyses, meta-regression), if done, indicating which were pre-specified.	8
**RESULTS**			
Study selection	17	Give numbers of studies screened, assessed for eligibility, and included in the review, with reasons for exclusions at each stage, ideally with a flow diagram.	9, Supplementary figure 1
Study characteristics	18	For each study, present characteristics for which data were extracted (e.g., study size, PICOS, follow-up period) and provide the citations.	9, Tables 1 and 2
Risk of bias within studies	19	Present data on risk of bias of each study and, if available, any outcome level assessment (see item 12).	12, Supplementary table 1
Results of individual studies	20	For all outcomes considered (benefits or harms), present, for each study: (a) simple summary data for each intervention group (b) effect estimates and confidence intervals, ideally with a forest plot.	10-13
Synthesis of results	21	Present results of each meta-analysis done, including confidence intervals and measures of consistency.	11
Risk of bias across studies	22	Present results of any assessment of risk of bias across studies (see Item 15).	11
Additional analysis	23	Give results of additional analyses, if done (e.g., sensitivity or subgroup analyses, meta-regression [see Item 16]).	NA
**DISCUSSION**			
Summary of evidence	24	Summarize the main findings including the strength of evidence for each main outcome; consider their relevance to key groups (e.g., healthcare providers, users, and policy makers).	14-15
Limitations	25	Discuss limitations at study and outcome level (e.g., risk of bias), and at review-level (e.g., incomplete retrieval of identified research, reporting bias).	16
Conclusions	26	Provide a general interpretation of the results in the context of other evidence, and implications for future research.	16
**FUNDING**			
Funding	27	Describe sources of funding for the systematic review and other support (e.g., supply of data); role of funders for the systematic review.	1

From: Moher D, Liberati A, Tetzlaff J, Altman DG, The PRISMA Group (2009). Preferred Reporting Items for Systematic Reviews and Meta-Analyses: The PRISMA Statement. PLoS Med 6(7): e1000097. doi:10.1371/journal.pmed1000097

For more information, visit: **www.prisma-statement.org**.

Appendix 2. Literature search strategy.

PubMed

(antigens, CD274[majr] OR B7DC[tiab] OR “B7-DC”[tiab] OR B7H1[tiab] OR “B7-H1”[tiab] OR BTLA[tiab] OR CD152[tiab] OR CD272[tiab] OR CD273[tiab] OR CD274[tiab] OR CD279[tiab] OR cell cycle checkpoints[majr] OR checkpoint[tiab] OR checkpoints[tiab] OR CTLA-4 antigen[majr] OR CTLA4[tiab] OR “CTLA-4”[tiab] OR “cytotoxic T-lymphocyte antigen 4”[tiab] OR “cytotoxic T-lymphocyte antigen4”[tiab] OR “immune stimulated”[tiab] OR “immune stimulating”[tiab] OR “immune stimulation”[tiab] OR “immune stimulations”[tiab] OR “immune stimulator”[tiab] OR “immune stimulators”[tiab] OR “immune stimulatory”[tiab] OR “lymphocyte attenuator”[tiab] OR “lymphocyte attenuators”[tiab] OR “PD ligand”[tiab] OR “PD ligands”[tiab] OR PD1[tiab] OR “PD-1”[tiab] OR PDCD1[tiab] OR PDCD1LG2[tiab] OR PDL1[tiab] OR “PD-L1”[tiab] OR PDL2[tiab] OR “PD-L2”[tiab] OR programmed cell death 1 ligand 2 protein[majr] OR “programmed cell death 1 ligand 1 protein”[tiab] OR “programmed cell death 1 ligand 2 protein”[tiab] OR programmed cell death 1 receptor[majr] OR “programmed cell death 1”[tiab] OR “programmed cell death ligand 1”[tiab] OR “programmed cell death ligand 2”[tiab] OR “programmed death 1”[tiab] OR “programmed death ligand”[tiab] OR “soluble PD-1”[tiab] OR “soluble PD1”[tiab] OR “soluble PD-L1”[tiab] OR “soluble PDL1”[tiab] OR “soluble programmed cell death receptor-1”[tiab] OR “soluble programmed cell death ligand-1”[tiab] OR “sPD-1”[tiab] OR “sPD1”[tiab] OR “sPD-L1”[tiab] OR “sPDL1”[tiab]) AND (sepsis[majr] OR sepsis[tiab] OR septic[tiab] OR septicemia[tiab] OR bacterial infections[majr] OR “bacterial infection”[tiab] OR “bacterial infections”[tiab] OR critical illness[majr] OR “critical illness”[tiab] OR “critical illnesses”[tiab] OR “critically ill”[tiab] OR critical care[majr] OR “critical care”[tiab] OR endotoxins[majr] OR endotoxin[tiab] OR endotoxins[tiab] OR endotoxemia[majr] OR endotoxemia[tiab] OR lipopolysaccharides[majr] OR lipopolysaccharide[tiab] OR lipopolysaccharides[tiab] OR “systemic inflammation”[tiab] OR "Bacterial Infections and Mycoses"[Mesh] OR "Virus Diseases"[Mesh]) AND ("Animals"[Mesh:NoExp] OR mouse[tiab] OR mice[tiab] OR rat[tiab] OR rats[tiab] OR dog[tiab] OR dogs[tiab] OR canine[tiab] OR “guinea pig”[tiab] OR “guinea pigs”[tiab] OR swine[tiab] OR pig[tiab] OR pigs[tiab] OR primate[tiab] OR primates[tiab]) AND (“3C10 monoclonal antibody”[tiab] OR abatacept[majr] OR abatacept[tiab] OR “anti-PD1”[tiab] OR “anti-PD-1”[tiab] OR “anti-PDL1”[tiab] OR “anti-PD-L1”[tiab] OR “anti-PDL2”[tiab] OR “anti-PD-L2”[tiab] OR atezolizumab[tiab] OR avelumab[tiab] OR durvalumab[tiab] OR ipilimumab[tiab] OR keytruda[tiab] OR lambrolizumab[tiab] OR nivolumab[tiab] OR opdivo[tiab] OR pembrolizumab[tiab] OR pidilizumab[tiab] OR tremelimumab[tiab] OR antagonists and inhibitors[subheading] OR antagonist[tiab] OR antagonists[tiab] OR inhibitor[tiab] OR inhibitors[tiab] OR inhibition[tiab] OR blockade[tiab] OR blocker[tiab] OR blockers[tiab])

EMBASE

('programmed death 1 ligand 1'/exp/mj OR b7dc:ti,ab OR 'b7-dc':ti,ab OR b7h1:ti,ab OR 'b7-h1':ti,ab OR btla:ti,ab OR cd152:ti,ab OR cd272:ti,ab OR cd273:ti,ab OR cd274:ti,ab OR cd279:ti,ab OR 'cell cycle checkpoint'/exp/mj OR checkpoint:ti,ab OR checkpoints:ti,ab OR 'cytotoxic t lymphocyte antigen 4'/exp/mj OR ctla4:ti,ab OR 'ctla-4':ti,ab OR 'cytotoxic t-

lymphocyte antigen 4':ti,ab OR 'cytotoxic t-lymphocyte antigen4':ti,ab OR 'immune stimulated':ti,ab OR 'immune stimulating':ti,ab OR 'immune stimulation':ti,ab OR 'immune stimulations':ti,ab OR 'immune stimulator':ti,ab OR 'immune stimulators':ti,ab OR 'immune stimulatory':ti,ab OR 'lymphocyte attenuator':ti,ab OR 'lymphocyte attenuators':ti,ab OR 'pd ligand':ti,ab OR 'pd ligands':ti,ab OR pd1:ti,ab OR 'pd-1':ti,ab OR pdcd1:ti,ab OR pdcd1lg2:ti,ab OR pdl1:ti,ab OR 'pd-l1':ti,ab OR pdl2:ti,ab OR 'pd-l2':ti,ab OR 'programmed death 1 ligand 2'/exp/mj OR 'programmed cell death 1 ligand 1 protein':ti,ab OR 'programmed cell death 1 ligand 2 protein':ti,ab OR 'programmed death 1 receptor'/exp/mj OR 'programmed cell death 1':ti,ab OR 'programmed cell death ligand 1':ti,ab OR 'programmed cell death ligand 2':ti,ab OR 'programmed death 1':ti,ab OR 'programmed death ligand':ti,ab OR 'soluble pd-1':ti,ab OR 'soluble pd1':ti,ab OR 'soluble pd-l1':ti,ab OR 'soluble pdl1':ti,ab OR 'soluble programmed cell death receptor-1':ti,ab OR 'soluble programmed cell death ligand-1':ti,ab OR 'spd-1':ti,ab OR 'spd1':ti,ab OR 'spd-l1':ti,ab OR 'spdl1':ti,ab) AND ('sepsis'/exp/mj OR sepsis:ti,ab OR septic:ti,ab OR septicemia:ti,ab OR 'bacterial infection'/exp/mj OR 'bacterial infection':ti,ab OR 'bacterial infections':ti,ab OR 'critical illness'/exp/mj OR 'critical illness':ti,ab OR 'critical illnesses':ti,ab OR 'critically ill':ti,ab OR 'intensive care'/exp/mj OR 'critical care':ti,ab OR 'endotoxin'/exp/mj OR endotoxin:ti,ab OR endotoxins:ti,ab OR 'endotoxemia'/exp/mj OR endotoxemia:ti,ab OR 'lipopolysaccharide'/exp/mj OR lipopolysaccharide:ti,ab OR lipopolysaccharides:ti,ab OR 'systemic inflammation':ti,ab OR 'infection'/exp/mj OR 'virus infection'/exp/mj) AND ('animal'/exp/mj OR mouse:ti,ab OR mice:ti,ab OR rat:ti,ab OR rats:ti,ab OR dog:ti,ab OR dogs:ti,ab OR canine:ti,ab OR 'guinea pig':ti,ab OR 'guinea pigs':ti,ab OR swine:ti,ab OR pig:ti,ab OR pigs:ti,ab OR primate:ti,ab OR primates:ti,ab) AND ('3c10 monoclonal antibody':ti,ab OR 'abatacept'/exp/mj OR abatacept:ti,ab OR 'anti-pd1':ti,ab OR 'anti-pd-1':ti,ab OR 'anti-pdl1':ti,ab OR 'anti-pd-l1':ti,ab OR 'anti-pdl2':ti,ab OR 'anti-pd-l2':ti,ab OR 'atezolizumab'/exp/mj OR atezolizumab:ti,ab OR 'avelumab'/exp/mj OR avelumab:ti,ab OR 'durvalumab'/exp/mj OR durvalumab:ti,ab OR 'ipilimumab'/exp/mj OR ipilimumab:ti,ab OR 'pembrolizumab'/exp/mj OR keytruda:ti,ab OR lambrolizumab:ti,ab OR 'nivolumab'/exp/mj OR nivolumab:ti,ab OR opdivo:ti,ab OR pembrolizumab:ti,ab OR 'pidilizumab'/exp/mj OR pidilizumab:ti,ab OR 'ticilimumab'/exp/mj OR tremelimumab:ti,ab OR antagonist:ti,ab OR antagonists:ti,ab OR inhibitor:ti,ab OR inhibitors:ti,ab OR inhibition:ti,ab OR blockade:ti,ab OR blocker:ti,ab OR blockers:ti,ab)

Scopus

TITLE-ABS (b7dc OR "B7-DC" OR b7h1 OR "B7-H1" OR btla OR cd152 OR cd272 OR cd273 OR cd274 OR cd279 OR checkpoint OR checkpoints OR ctla4 OR "CTLA-4" OR "cytotoxic T-lymphocyte antigen 4" OR "cytotoxic T-lymphocyte antigen4" OR "immune stimulated" OR "immune stimulating" OR "immune stimulation" OR "immune stimulations" OR "immune stimulator" OR "immune stimulators" OR "immune stimulatory" OR "lymphocyte attenuator" OR "lymphocyte attenuators" OR "PD ligand" OR "PD ligands" OR pd1 OR "PD-1" OR pdcd1 OR pdcd1lg2 OR pdl1 OR "PD-L1" OR pdl2 OR "PD-L2" OR "programmed cell death 1 ligand 1 protein" OR "programmed cell death 1 ligand 2 protein" OR "programmed cell death 1" OR "programmed cell death ligand 1" OR "programmed cell death ligand 2" OR "programmed death 1" OR "programmed death ligand" OR "soluble PD-

1" OR "soluble PD1" OR "soluble PD-L1" OR "soluble PDL1" OR "soluble programmed cell death receptor-1" OR "soluble programmed cell death ligand-1" OR "sPD-1" OR "sPD1" OR "sPD-L1" OR "sPDL1") AND TITLE-ABS (sepsis OR septic OR septicemia OR "bacterial infection" OR "bacterial infections" OR "critical illness" OR "critical illnesses" OR "critically ill" OR "critical care" OR endotoxin OR endotoxins OR endotoxemia OR lipopolysaccharide OR lipopolys accharides OR "systemic inflammation") AND TITLE-ABS (mouse OR mice OR rat OR rats OR dog OR dogs OR canine OR "guinea pig" OR "guinea pigs" OR swine OR pig OR pigs OR primate OR primates) AND TITLE-ABS ("3C10 monoclonal antibody" OR abatacept OR "anti-PD1" OR "anti-PD-1" OR "anti-PDL1" OR "anti-PD-L1" OR "anti-PDL2" OR "anti-PD-L2" OR atezolizumab OR avelumab OR durvalumab OR ipilimumab OR keytruda OR la mbrolizumab OR nivolumab OR opdivo OR pembrolizumab OR pidilizumab OR tremelim umab OR antagonist OR antagonists OR inhibitor OR inhibitors OR inhibition OR blocka de OR blocker OR blockers)

Web of Science

TOPIC: (B7DC OR B7-DC OR B7H1 OR B7-H1 OR BTLA OR CD152 OR CD272 OR CD273 OR CD274 OR CD279 OR checkpoint OR checkpoints OR CTLA4 OR CTLA-4 OR cytotoxic T-lymphocyte antigen 4 OR cytotoxic T-lymphocyte antigen4 OR immune stimulated OR immune stimulating OR immune stimulation OR immune stimulations OR immune stimulator OR immune stimulators OR immune stimulatory OR lymphocyte attenuator OR lymphocyte attenuators OR PD ligand OR PD ligands OR PD1 OR PD-1 OR PDCD1 OR PDCD1LG2 OR PDL1 OR PD-L1 OR PDL2 OR PD-L2 OR programmed cell death 1 ligand 1 protein OR programmed cell death 1 ligand 2 protein OR programmed cell death 1 OR programmed cell death ligand 1 OR programmed cell death ligand 2 OR programmed death 1 OR programmed death ligand OR soluble PD-1 OR soluble PD1 OR soluble PD-L1 OR soluble PDL1 OR soluble programmed cell death receptor-1 OR soluble programmed cell death ligand-1 OR sPD-1 OR sPD1 OR sPD-L1 OR sPDL1) AND TOPIC: (sepsis OR septic OR septicemia OR bacterial infection OR bacterial infections OR critical illness OR critical illnesses OR critically ill OR critical care OR endotoxin OR endotoxins OR endotoxemia OR lipopolysaccharide OR lipopolysaccharides OR systemic inflammation) AND TOPIC: (mouse OR mice OR rat OR rats OR dog OR dogs OR canine OR guinea pig OR guinea pigs OR swine OR pig OR pigs OR primate OR primates) AND TOPIC:(3C10 monoclonal antibody OR abatacept OR anti-PD1 OR anti-PD-1 OR anti-PDL1 OR anti-PD-L1 OR anti-PDL2 OR anti-PD-L2 OR atezolizumab OR avelumab OR durvalumab OR ipilimumab OR keytruda OR lambrolizumab OR nivolumab OR opdivo OR pembrolizumab OR pidilizumab OR tremelimumab OR antagonist OR antagonists OR inhibitor OR inhibitors OR inhibition OR blockade OR blocker OR blockers)

Appendix 3: Effect of checkpoint inhibitor treatment on serum and tissue cytokines, immune cell populations, and apoptosis. Refer to Table 5 for detailed results.

Five experiments reported the effects of CPI treatment on serum cytokine levels. Anti-PD-L1 increased serum TNFα and IL-6 and decreased IL-10 on D+1 after CLP and decreased IL-6, IL-10, MIP-2, KC and IL-17 on D+2 after ID *P. aeruginosa* ($p \leq 0.05$); anti-PD-1 and anti-CTLA-4 had no significant effect on IL-6, IL-10, TNFα or IFNγ on D+2 after CLP (all p=ns); and anti-BTLA increased serum MIP-2 on D+1 after CLP ($p<0.05$) but had no significant effect (p=ns) on TNFα, IL-1β, IL-6, IL-10, IL-12, KC, or MCP-1. In this latter study, anti-BTLA increased peritoneal lavage TNFα, IL-10, IL-12, KC, MIP-2, and MCP-1 ($p \leq 0.05$) but did not significantly alter IL-1β and IL-6 on D+1.

Eight experiments reported the effects of CPI treatment on *ex-vivo* immune cell cytokine or nitric oxide (NO) production. Anti-PD-1 increased CD3/CD28-stimulated splenocyte production of IL-6 but not TNFα, IL-10 and INFγ at D+2 after CLP in one experiment, increased CD3/CD28-stimulated splenocyte production of IFNγ on D9 after CLP and subsequent *C. albicans* infection, and increased splenic NK, CD4 and CD8 intracellular IFNγ levels on D+9 after CLP and subsequent *C. albicans* infection; anti-PD-L1 decreased heat killed *L. monocytogenes* stimulated splenocyte TNFα, IL-12p40 and NO and NK cell IFNγ production on D+3 after IV *L. monocytogenes* challenge, increased spleen and lymph node CD8 IFNγ, decreased lymph node CD4 IFNγ but did not alter spleen CD4 IFNγ on D+2 after ID *P. aeruginosa*, and did not alter CD3/CD28-stimulated splenocyte IFNγ on D+9 after CLP and subsequent *C. albicans* infection; anti-CTLA-4 did not alter CD3/CD28-stimulated splenocyte

production of TNFα, IL-6 or IL-10 on D+2 after CLP; and anti-BTLA increased LPS-stimulated macrophage production of TNFα and MIP-2 on D+1 after CLP.

Nine experiments reported the effects of CPI treatment on changes in immune cell numbers and phenotype. Anti-PD-L1 treatment decreased splenic *L. monocytogenes*-specific and IFNγ-producing CD8 cells on D+7 after IV *L. monocytogenes*, increased total cell numbers, CD3 and CD19 cell numbers in blood, spleen and thymus on D+1, increased IFNγ producing CD4 and CD8 cells and increased MHCII expression on DCs and macrophages on D+9 after CLP and subsequent fungal challenge and increased spleen and lymph node CD4 and CD8 cell numbers and CD28 expression, CD19+ B-cells in lymph nodes, but not in spleen, on D+2 after ID *P. aeruginosa* (all $p \leq 0.05$). Anti-PD-1 treatment increased splenic CD4 and CD8 cells, B-cells, NK cells, and dendritic cells on D+2 after CLP, increased macrophage and DC MHCII expression on D+9 after CLP followed by fungal infection, and increased lymph node CD4/CD28 and CD8/CD28 cells (all $p \leq 0.05$) but did not significantly alter splenic and lymph node CD4/CD28 and CD8/CD28, macrophages, or dendritic cells on D+9 after CLP and subsequent *C. albicans* challenge (p=ns). Anti-CTLA-4 treatment did not significantly alter total splenocyte CD4, CD8 numbers or naïve, or effector or central memory phenotype cells at D+7 following CLP. Finally, anti-BTLA treatment increased total peritoneal leukocytes and F4/80 CD11c and Grl+ cells on D+1 after CLP ($p<0.05$).

Seven experiments reported the effects of CP[illegible]n apoptosis. Anti-PD-L1 treatment did not affect the frequency of Annexin V positive CD8 cells on D+1, decreased splenic and thymus lymphocyte apoptosis on D+1 after CLP and decreased splenic CD4 and CD8 cell apoptosis on D+2 and D+3 after CLP (all $p \leq 0.05$); anti-PD-1 treatment decreased splenic CD3 T[illegible]l apoptosis on D+2 after CLP ($p<0.05$); anti-CTLA-4 treatment decreased splenic CD4 and CD8

cell apoptosis on D+2 after CLP ($p<0.05$); and anti-BTLA did not significantly alter peritoneal total cell or macrophage apoptosis on D+1 after CLP (p=ns).

Supplementary Figure Legends

Supplementary Figure 1. Flow chart of literature search and study selection.

Supplementary Figure 2. Funnel plot of odds ratio (OR) of survival. Publication bias was assessed by funnel plot and Egger's regression. This assessment suggested that the overall survival results were not subject to publication bias (Egger's statistic p=0.96).

Supplementary Table 1. Effect of bacterial challenge on the checkpoint molecules targeted in analyzed experiments

Author/year	Exp ID	Checkpoint Molecule Target	Bacterial Challenge	Infection Site	Summary of Effect of Challenge on Targeted Molecule in Animals not Receiving Checkpoint Inhibitor Treatment
Seo 2008	1	PD-L1	*L. monocytogenes*	IV	Splenic CD4, CD8, NK, and macrophage PD-L1 expression increased on D+3 compared to noninfected controls (no p-value reported)
Brahmamdam 2010	1	PD-1	CLP	IP	Splenic CD4 and CD8 PD-1 expression increased on D+2,+4,+7 compared to noninfected controls^^
Zhang* 2010	1	PD-L1	CLP	IP	Circulating CD19+ B cell and CD11b+ monocyte PD-1 and PD-L1 and CD3+ PD-1 expression increased on D+1 compared to noninfected controls^^, but CD3+ PD-L1 expression was not different (p=ns)
	2	PD-L1	CLP	IP	
Inoue** 2011	1	CTLA-4	CLP	IP	Splenic CD4+ CTLA-4 expression increased on D+1,+4,+7 ^, Splenic CD8+ CTLA-4 expression increased on D+4 and +7^, Splenic Treg CTLA-4 expression increased at D+1 and +7^ compared to noninfected controls
	2	CTLA-4	CLP	IP	
	3	CTLA-4	CLP	IP	
	4	CTLA-4	CLP (*Candida* D+4) [##]	IP	NR
Kobayashi 2013	1	BTLA	LPS	IV	NR
Chang 2013	1	PD-1	CLP (*Candida* D+3)	IP and IV	Splenic CD4 + T cell PD-1 expression increased on D+3 (before Candida) and D+5,+9,+12 compared to baseline^. Splenic CD8+ T cell PD-1 expression increased on D+3 and +5 compared to baseline^
	2	PD-L1	CLP (*Candida* D+3)	IP and IV	
	3	CTLA-4	CLP (*Candida* D+3)	IP and IV	NR
Shindo 2015	1	PD-1	CLP (*Candida* D+3)	IP and IV	NR
Cheng 2016	1	BTLA	CLP	IP and IV	Peritoneal F4/80 macrophage and CD11c DC BTLA expression increased and circulating $CD115^+$ DC BTLA expression decreased on D+1 compared to noninfected controls^
Shindo 2017	1	PD-L1	CLP (*Candida* D+3)	IP and IV	Splenic NK, NKT, and CD4 PD-L1 expression increased^^ on D+7 compared to noninfected controls but splenic CD8, macrophage, and B cell PD-L1 expression not different (p=ns)
Deng[#] 2018	1	PD-L1	CLP	IP	NR
	2	PD-L1	CLP	IP	NR
Patil 2018	1	PD-L1	*P. aeruginosa*	Dermal	Splenic CD11c DC and F4/80 macrophage PD-L1 expression increased on D+2 compared to noninfected controls^ but splenic Ly6c monocytes and CD4 and CD8 PD-1 expression not different (p=ns)
	2	PD-L1	*S. aureus*	IV	NR

*exp 1 treatment administered at D-1 and exp 2 treatment administered at D0; **exp 1 administered 50μg and exp 2 administered 200μg anti-CTLA-4 in CD-1 mice, exp 3 administered 50μg anti-CTLA-4 in C57BL6 mice; [#]exp 1 performed in C57BL6J mice and exp 2 in $Bmal1^{Mye-/-}$ mice; [##] intravenous *Candida* challenge 4 days following CLP; ^p<0.05 ; ^^p≤0.01

Exp ID – number assigned to the experiment(s) providing survival data in each study; PD-1 - programmed cell death 1; PD-L1 – programmed cell death ligand-1; CTLA-4 – cytotoxic T lymphocyte associated protein-4; BTLA – B and T lymphocyte attenuator; CLP – cecal ligation and puncture; DC – dendritic cell; D – day; IV - intravenous; IP - intraperitoneal; NR – not reported

Supplementary Figure 1

Literature Search Results

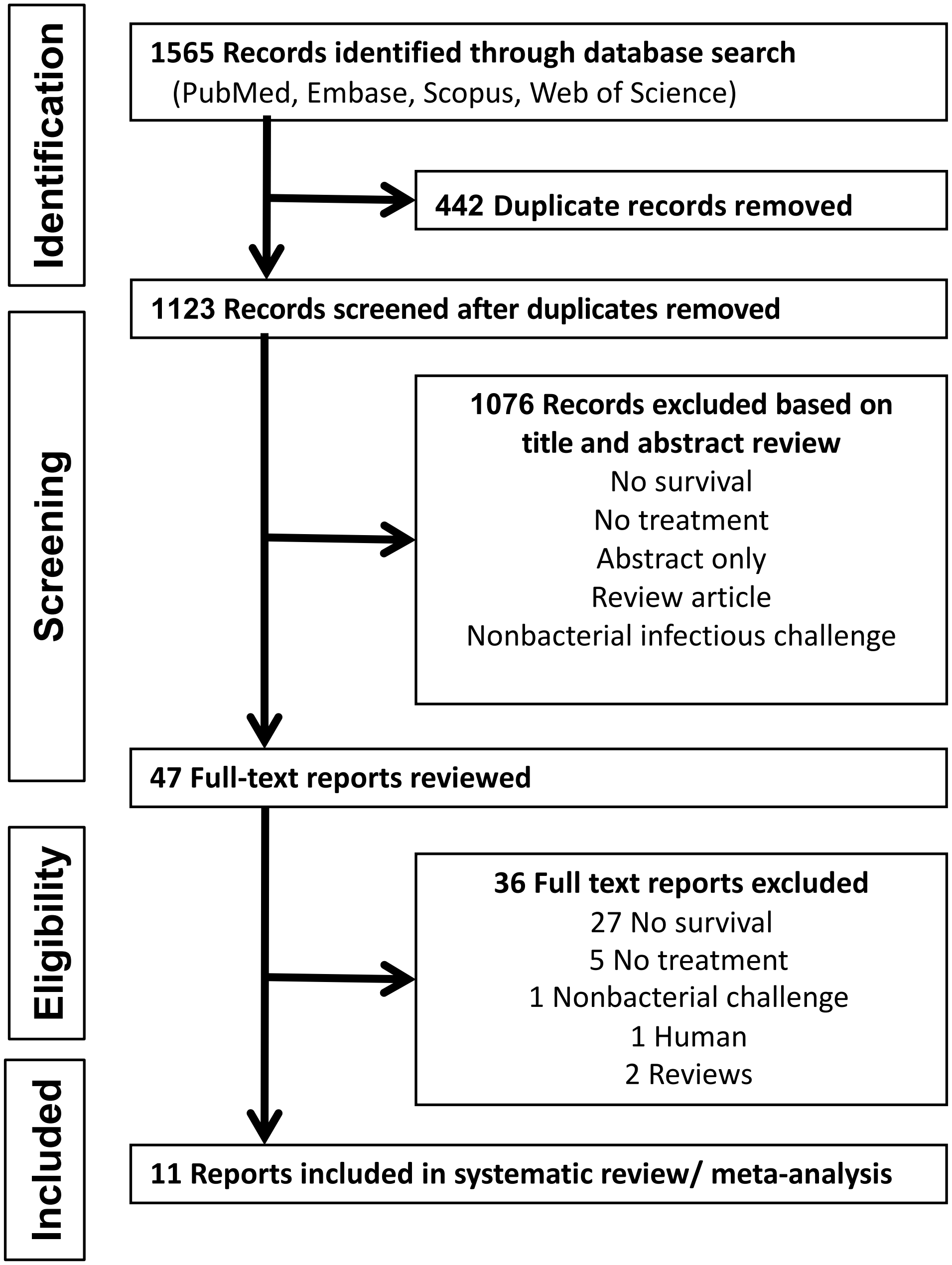

Supplementary Figure 2

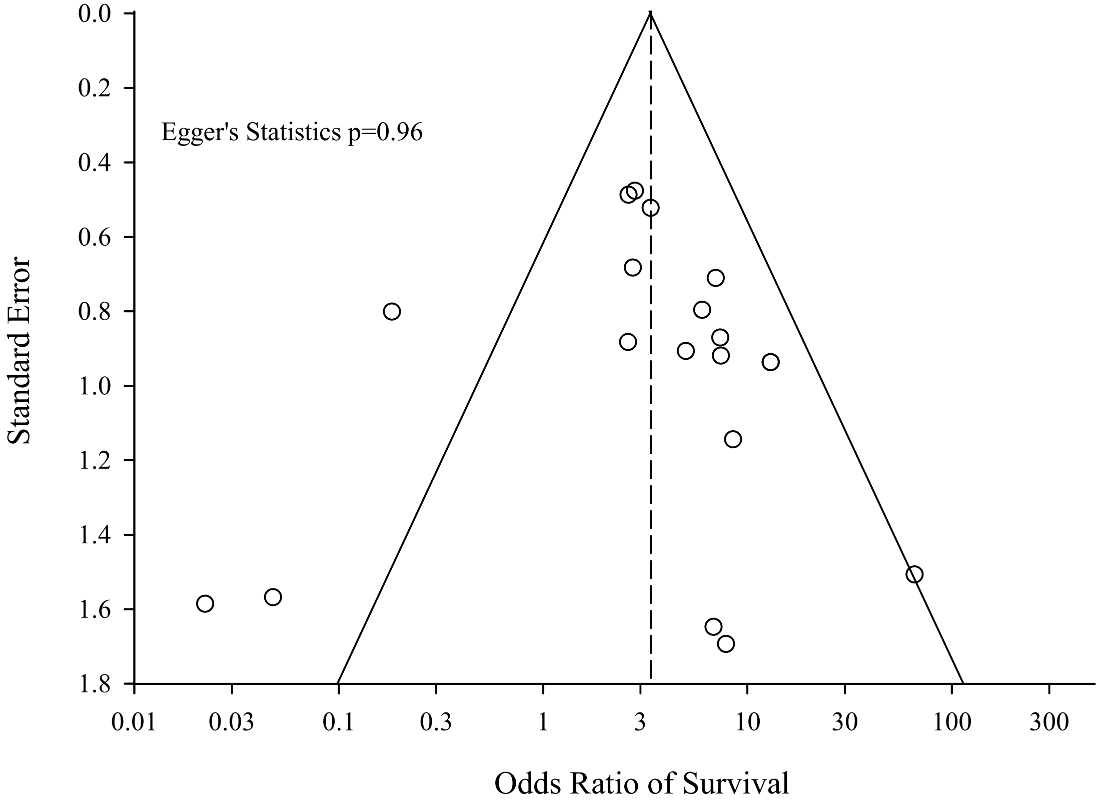

Supplement: Supplementary file 1 — Additional file 1. Appendix 1. Prisma checklist. Appendix 2. Literature search strategy. Appendix 3. Effect of checkpoint inhibitor treatment on serum and tissue cytokines, immune cell populations, and apoptosis. Table 1. Effect of bacterial challenge on the checkpoint molecules targeted in analyzed experiments. Figure 1. Flow chart of literature search and study selection. Figure 2. Funnel plot of odds ratio (OR) of survival. [file 40635_2019_290_MOESM1_ESM.pdf]
